# Acidic extracellular microenvironment and cancer

**DOI:** 10.1186/1475-2867-13-89

**Published:** 2013-09-03

**Authors:** Yasumasa Kato, Shigeyuki Ozawa, Chihiro Miyamoto, Yojiro Maehata, Atsuko Suzuki, Toyonobu Maeda, Yuh Baba

**Affiliations:** 1 Department of Oral Function and Molecular Biology, Ohu University School of Dentistry, 963-8611, Koriyama, Japan; 2 Department of Oral Maxillofacial Surgery, Kanagawa Dental University Graduate School of Dentistry, Yokosuka, Japan; 3 Department of Oral Science, Kanagawa Dental University Graduate School of Dentistry, Yokosuka, Japan; 4 Department of General Clinical Medicine, Ohu University School of Dentistry, Koriyama, Japan

**Keywords:** Acidic microenvironment, Cancer, Malignant phenotype

## Abstract

Acidic extracellular pH is a major feature of tumor tissue, extracellular acidification being primarily considered to be due to lactate secretion from anaerobic glycolysis. Clinicopathological evidence shows that transporters and pumps contribute to H^+^ secretion, such as the Na^+^/H^+^ exchanger, the H^+^-lactate co-transporter, monocarboxylate transporters, and the proton pump (H^+^-ATPase); these may also be associated with tumor metastasis. An acidic extracellular pH not only activates secreted lysosomal enzymes that have an optimal pH in the acidic range, but induces the expression of certain genes of pro-metastatic factors through an intracellular signaling cascade that is different from hypoxia. In addition to lactate, CO_2_ from the pentose phosphate pathway is an alternative source of acidity, showing that hypoxia and extracellular acidity are, while being independent from each other, deeply associated with the cellular microenvironment. In this article, the importance of an acidic extracellular pH as a microenvironmental factor participating in tumor progression is reviewed.

## Introduction

The extracellular pH (pH_*e*_) of tumor tissues is often acidic [1], and acidic metabolites, *e.g.* lactic acid caused by anaerobic glycolysis in hypoxia, seem to be the main cause. Accumulating evidence shows that an acidic microenvironment is a regulator of cellular phenotype. Whereas Na^+^-HCO_3_^-^ co-transporter and Cl^-^/HCO_3_^-^ exchanger contribute a fall in intracellular pH, the Na^+^/H^+^ exchanger (NHE) [2], the H^+^-lactate co-transporter, monocarboxylate transporters (MCTs), and the H^+^-ATPase (H^+^ pump) are responsible for the secretion of H^+^[3]. Because carbonic anhydrase (CA) is widely distributed and can form H^+^ by catalyzing hydration of CO_2_, an excess amount of CO_2_ production through the pentose phosphate pathway in tumor cells is an alternative cause of a lower pH [4]. Acidic pH_*e*_ increases not only the activation of some lysosomal enzymes with acidic optimal pH, but also the expression of some genes involved with pro-metastatic factors. When melanoma cells pretreated with an acidic medium were injected into the tail vein of mice, a significantly higher frequency of them metastasized to the lungs [5]. Thus, an acidic microenvironment is closely associated with tumor metastasis.

Acidity is found at the surface of skin and in inflammatory sites. It is also associated with bone resorption. Thus, an acidic microenvironment plays a role of homeostasis and the immune defense system. We will review the roles of acidic pH_*e*_ in tumor progression along with other physiological and pathological conditions.

### Lactate and tumor

The “Warburg effect” is a well-accepted theory that says that tumors tend to produce lactate by using the anaerobic glycolytic pathway, even in the presence of sufficient oxygen, rather than oxidative phosphorylation for energy production [1]. High lactate levels indicate metastases, tumor recurrence, and prognosis in some cancer patients [6-9]. In the molecular mechanism relating to these clinical contributions, lactate from tumor cells contributes to their immune escape. High lactate secretion from tumor cells inhibits its export from T cells, thereby disturbing their metabolism and function [10]. Tumor-derived lactate affects inflammation and immune deficiency of tumor cells. Lactate itself functions as an intrinsic inflammatory mediator that increases interleukin (IL)-17A production by T-cells and macrophages, resulting in the promotion of chronic inflammation in tumor microenvironments [11]. Lactate inhibits dendritic cell activation during antigen-specific autologous T-cell stimulation [12]. It also enhances the motility of tumor cells and inhibits monocyte migration and cytokine release [13]. It can contribute to angiogenesis through induction of IL-8 via nuclear factor-κB (NF-κB) [14] and induction of vascular endothelial growth factor (VEGF/VEGF-A) via hypoxia-inducible transcription factor (HIF)-1 [15]. Furthermore, lactate production contributes to radio-resistance of tumors due to its antioxidant properties [16].

Inhibition of the lactate transporter has been considered a potential new therapeutic strategy. For example, α-cyano-4-hydroxycinnamate, a specific inhibitor of the lactate transporter MCT1, suppresses tumor angiogenesis [17]. Quercetin (CYP2C9), which is an inhibitory flavonoid, inhibits lactate transport and acts as a hyperthermic sensitizer of HeLa cells [18].

### Appearance of acidic microenvironments under physiological and pathological conditions

An oncogenic transformation assay by oncogenic-virus infection shows that lactate production is correlated with an increase in the number of transformed foci by viral infection in a presence of 5% CO_2_ in 95% air [19]. Since high lactate corresponds to a high proton concentration, an acidic pH_*e*_ is a major feature of the solid tumor tissue [1,20-22]. Lactic acid is a product of the anaerobic glycolysis including the activity of lactate dehydrogenase (LDH) 5 that generates lactic acid from pyruvate and the expression of which has been strongly associated with the poor prognosis of patients with non-small cell lung [23,24] and colorectal cancers [25-27].

CO_2_ is a major source of acid in glycolytically impaired mice [4]. The pentose phosphate pathway is seen as a major productive pathway for CO_2_ which can be processed to H^+^ and HCO_3_^-^ by the catalytic activity of CA. In osteoclasts, CA II, a CA isozyme, is a major enzyme producing H^+^ to decalcify bone hydroxyapatite. Osteoclasts secrete H^+^ and create an acidic microenvironment below pH 5.5, which is critical for the bone resorption [28,29] and the proton can be secreted through H^+^-ATPase [30]. Induction of CA II expression itself is also induced by an acidic pH_*e*_[31]. Thus, secretion of acidic metabolites and/or the pentose phosphate pathway-mediated CO_2_ production, and CA-mediated production of H_2_CO_3_ form acidic microenvironments.

Extracellular acidity is a pathological feature of inflammation [32] and solid tumor tissue [1,20-22]. Acidity in inflammatory tissue is due to production of proton from macrophages, whereas tumor tissue acidity is due to acidic metabolites, *e.g.*, lactate, caused by anaerobic glycolysis under the hypoxia [20-22,33]. The acidic microenvironment acts as a trigger for pain in both inflammation [34,35] and in cancer patients [36].

Ovarian cancer G-protein-coupled receptor 1 (OGR1), a receptor for sphingosylphosphorylcholine, and GPR4, a close relative of OGR1, also act as a proton-sensing receptor in osteosarcoma cells and primary human osteoblast precursors [37]. OGR1 (GPR68) stimulates cyclooxygenase-2 expression and prostaglandin (PG) E_2_ production in response to acidic pH_*e*_ in a human osteoblastic cell line [38]. Because PGE_2_ is involved in osteoclastic differentiation of precursor cells [39], inhibition of the OGR1 signaling negatively regulates osteoclastogenesis [40]. Another type of G-protein-coupled receptor, TDAG8 (GPR65), also senses pH_*e*_[41,42].

Breast cancer frequently metastasizes to bone. Osteoclasts can be activated by breast cancer-derived H^+^ such that osteolysis occurs when cancer cells metastasize to bone [36]. During this process, patients feel pain through acid-sensing ion channels (ASIC) 1a, 1b and 3 [36,43,44].

An acidic pH_*e*_ is also found in the epidermis and plays an important protective role against bacterial infection [45-47]. Using the conditional knockout (KO) mice for focal adhesion kinase (FAK) in keratinocytes, Ilic et al. [47] showed that the stratum corneum pH_*e*_ gradient of keratinocytes in these mice had significantly more neutral pH values, and that NHE1 failed to localize to the plasma membrane [47]. Thus, FAK controls pH-dependent epidermal barrier homeostasis by regulating actin-directed NHE1 plasma membrane localization [47].

Lung liquid is acidic [48], which is worse in patients with cystic fibrosis [49], although the airway pH is not known for certain because different detecting methods have been used [50].

### CA expression in cancer

CA isoforms are associated with tumor malignancy, including CA I [51], CA II [51,52], CA IX [53,54], CA XII [55], and CA XIII [56]. Among them, CA IX in particular has been well studied in association with hypoxia and tumor survival through regulating intracellular pH [53,57]. In ovarian cancer, high expression of CA IX with a concomitant increase in VEGF-A is associated with overall survival rates positively [58]. Overexpression of CA IX increases tumor cell migration and invasion [59]. CA inhibitor suppresses invasion of renal cancer cells *in vitro*[60]. Based on the accumulated evidence, a new therapeutic strategy targeting CA has been considered [61-63].

### Acidic pH_*e*_ activates proteinase activity and induces gene expression

Acidic pH_*e*_ activates some proteinases. Although caries is due to some bacterial acidic metabolites, Tjäderhane et al. [64] found that host-derived pro-matrix metalloproteinase-9 (proMMP-9), proMMP-2 and proMMP-8 in saliva could be activated by acid, and thereby suggested that these MMPs contribute to the disruption of dentin in caries. Alternatively, host derived proMMP-9 could be activated in the stomach, and this suggests it functions as a digestive enzyme for collagenous foods [65,66]. Activation of proMMP-9 by an acidic pH_*e*_ also occurs in a human melanoma model [67].

Lysosomal enzymes have an acidic optimal pH. Some tumor cells have the ability to secrete them, such as cathepsin B and cathepsin L [5]. Cathepsin K plays an important role in osteoclast-mediated bone resorption [68,69]; its inhibition prevents breast cancer-induced osteolysis and skeletal tumor burden [70]. Thus, osteoclast-mediated acidic pH_*e*_ leads to mineral dissolution and activation of cathepsins to digest bone matrix, such as type I collagen. Podgorski et al. [71] reported that SPARC/osteonectin, a major non-collagenous protein in bone, is digested by cathepsin K and its fragments are associated with bone-metastasis. Another lysosomal enzyme, heparanase, has an acidic optimal pH; it degrades heparan sulfate in the basement membrane and contributes to tumor invasion and metastasis [72,73].

Also, acidic microenvironments affect the expression of some genes, such as MMP-9 [74,75] and acidic sphingomyelinase in mouse B16 melanoma [74], platelet-derived endothelial cell growth factor (thymidine phosphorylase) in human breast cancer cells [76], the inducible isoform of nitric oxide synthase (iNOS) in macrophages [77], VEGF-A in glioma [78] and glioblastoma [79] cells, and IL-8 expression in human pancreatic adenocarcinoma [80-82] and ovarian carcinoma cells [83].

### Acidic pH_*e*_ signal transduction pathway

Thus, although acidic pH_*e*_ occurs in several physiological and pathological conditions, information on its signaling remains limited. Transcription factors AP-1 and NF-κB, independent of hypoxia, have important roles in the acidic pH_*e*_-induced expression of VEGF-A [78,84] and IL-8 [80-83,85]. p38 mitogen-activated protein kinase (MAPK) is involved in acidic pH_*e*_ signaling that induces IL-8 [85].

We also found involvement of phospholipase D (PLD) in the acidic pH_*e*_-intracellular signaling to induce MMP-9 production [75,86]. Acidic pH_*e*_-induced PLD activation was prolonged for at least for 24 h, different from general growth factor signaling. Inhibition of PLD activity by 1-butanol and Myr-ARF6 suppresses acidic pH_*e*_-induced MMP-9 expression [87]. Acidic pH_*e*_ increases the steady-state levels of phosphorylated ERK1/2 and p38, and PLD inhibitors prevent these increases. Using 5′-deleted constructs of the MMP-9 promoter, we found that the acidic pH_*e*_-responsive region was located at nucleotides -670 to -531, a region containing the NF-κB binding site. A mutation in the NFκB binding site reduced acidic pH_*e*_-induced MMP-9 promoter activity, and NF-κB activity was induced by acidic pH_*e*_. Pharmacological inhibitors specific for MEK1/2 (PD098059) and p38 (SB203580) attenuated acidic pH_*e*_-induced NF-κB activity and MMP-9 expression. The data suggest that PLD, MAPKs including ERK 1/2 and p38, and NF-κB mediate acidic pH_*e*_ signaling thereby inducing MMP-9 expression. Activation of ERK1/2 and p38, followed by the NF-κB axis, which is stimulated by tumor necrosis factor-α (TNF-α), also occurs in cholangiocarcinoma [88]. This suggests that acidic pH_*e*_ signaling is, at least in part, the signaling pathway for TNF-α. However, it has been reported that acidic pH_*e*_ activates p38, but not ERK1/2, in T-cell receptor signaling in Jurkat cells [89]. This may be cell-type specific. In a further contribution dealing with the intracellular substances of acidic pH_*e*_, we have found that calcium influx triggers acidic pH_*e*_-induced PLD activation and that acidic sphingomyelinase mediates acidic pH_*e*_ signaling to activate NF-κB independently of the PLD-MAPK pathway [74].

OGR1 stimulates cyclooxygenase-2 expression and PGE_2_ production in response to an acidic pH_*e*_ in a human osteoblastic cell line through G(q/11)/phospholipase C/protein kinase C pathway [38] and in human aortic smooth muscle cells through the phospholipase C/cyclooxygenase/PGI_2_ pathway [90].

Acidic pH directly affects transcription factor activity; DNA binding activity of the transcription factor, SP1, is enhanced by intracellular acidic pH [91]. Intracellular pH is maintained a constitutively neutral state but known to become transiently acidic when pH_*e*_ decreases to acidic. Therefore an acidic pH can activate SP1.

### Acidic pH_*e*_ stimulates disruption of adherence junctions

When tumor cells move into their surrounding tissue, cell-cell junctions become dissociated. Acidic pH disrupts adherence junction by Src activation, resulting in E-cadherin degradation through the protein kinase Cδ pathway [92,93]. Acidic pH_*e*_ also induces motility of tumor cells, and inhibits monocyte migration and cytokine release [13].

### Acidic pH_*e*_ stimulates metastatic potential

Brockton et al. [54] have shown that high stromal CA IX expression is associated with nodal metastasis. The high activity produces an acidic microenvironment that leads to increased metastatic ability of the tumor cells. We have reported that induction rate of MMP-9 secretion correlates with metastatic potential of mouse B16 melanoma clones, and an acidic pH_*e*_ stimulates invasion through a type-IV collagen barrier [75,86]. In human melanoma models, an acidic pH_*e*_ increases both migration and invasiveness *in vitro,* accompanied by MMP-9 activation [67]. NHE1 is also associated with the metastatic ability of tumor cells; it is accumulated in leading edge of the cell and is activated by CD44 (a hyaluronan (HA) receptor) -binding to HA [94]. Because HA directs membrane-type 1 matrix metalloproteinase (MT1-MMP) to the invasion front (invadopodia) [95,96], NHE1 might interact with MT1-MMP through CD44 at an acidic pH_*e*_[97,98].

Pretreatment of the tumor cells in an acidic medium induces production of proteinases (MMPs and cathepsins) and proangiogenic factors (VEGF-A and IL-8) and promotes experimental metastasis to the lung after injection into the tail vein of nude mice [5]; elevation of pH by one unit following injection of sodium bicarbonate prevents spontaneous metastases [99]. Furthermore, using P-31 magnetic resonance spectroscopic evaluation, it was found that acidic pH_*e*_ in spontaneous soft tissue sarcomas predicts metastasis in dogs [100].

### Acidic pH_*e*_ sensing systems

ASICs are voltage-independent and proton-activated channels found in tumor cells and associated tumor malignancy [101]. Transient receptor potential (TRP) V isoforms, TRPV1, TRPV5 and TRPV6, also act as acid-sensitive channels [102,103]. ERK1/2 plays as a downstream target of ASICs and TRPVs [104-106]. Another subfamily of TRP, TRPM7 has proton conductivity [107]. TRPM7 regulates EGF signaling to induce STAT3 activation and vimentin expression during epithelial-mesenchymal transition [108]. OGR1 also acts as a proton-sensing receptor, stimulating inositol phosphate formation [37].

### pH_*e*_ gradient formation by H^+^ pumps and exchangers

NHE1 accumulates at the leading edge to make a pH_*e*_ gradient associated with cell migration [109]. The Rho-ROCK pathway contributes to NHE1 activation and focal adhesions [110,111]. Protons stabilize the collagen–α2β1 integrin bond, but alkalosis, a lack of protons or an inhibited NHE activity, prevents adhesion [112]. Furthermore, the cell forms an individual pH_*e*_ gradient to facilitate movement: *i.e.* at leading edge or invadopodia, cells preferentially attach to the substrate due to the acidic pH_*e*_ induced by NHE1, while cell-matrix interaction at the rear end is weak due to a mid-alkaline pH_*e*_[113]. Mutation studies clearly showed that downregulation of NHE1 function suppresses cell polarity, migration, and invasion through matrigel™ [111]. Inhibition of NHE1 activity by HOE642 (cariporide) reduced migration and adhesion activities [109].

To secrete acidic metabolites, NHE1 and the H^+^-lactate co-transporter are involved [114]. H^+^-ATPase (the H^+^ pump) and cell surface ATP synthase also play a role in extracellular acidification [115,116], thereby contributing to tumor metastasis [3]. Therefore, inhibition of the H^+^ pump can be a new strategy for cancer treatment [117-119]. Angiostatin has anti-tumor efficacy by inhibiting cell surface ATP synthase activity through binding its β subunit [116]. In particular, treatment of the cells with angiostatin proved more cytotoxicity at an acidic pH_*e*_ than a neutral pH_*e*_.

### Drug efficacy and acidic pH_*e*_

Two analogues of camptothecin (CPT), topotecan (TPT) and irinotecan (CPT-11), have significant anti-tumor activity in the clinic, although their abilities depend on the CPT E ring lactone, which forms an inactive hydroxy acid at physiological pH. The reaction is reversible at an acidic pH_*e*_, which provides a rationale for selectivity because many solid tumors, while creating an acidic extracellular environment, maintain a normal intracellular pH [120]. An acidic pH_*e*_ inhibits cellular uptake of mitoxantrone and topotecan, so that elevation of pH_*e*_ in tumor tissue enhances those drugs’ efficacy [120,121]. Because the buffer action is weaker in tumor tissue than normal tissue, NaHCO_3_ has much potential to raise pH_*e*_ relatively specifically in tumor tissue [122,123]. Acidic pH_*e*_ also plays a role in the resistance of tumor cells to drugs by increasing the expression of p-glycoprotein, thereby increasing drug efflux [124,125]. Recently, an acidic pH_*e*_-specific drug-releasing system has been developed [126,127]. A novel polymeric micelle constituted of 2 block copolymers of poly (L-lactic acid)-b-poly(ethylene glycol) b-poly (L-histidine) - TAT (transactivator of transcription) and poly(L-histidine)-b-poly(ethylene glycol) increases the cytotoxicity of doxorubicin in several multidrug-resistant tumor cell lines [127]. To measure pH_*e*_, a magnetic resonance image technique has been developed using acidic pH_*e*_ specific probes [128,129]. Thus, clinicians should pay attention to tumor pH_*e*_ in selecting drugs and helping to maximize their chemotherapeutic action. Vasodilating drugs, such as hydralazine and captopril, inhibit tumor growth rate *in vivo* by reducing tumor blood flow [130]. Although the reduction in tumor growth by those drugs also reduces the oxygen supply, it reduces pH_*e*_. In patients given vasodilating drugs, anti-tumor drugs with weak acidic p*K*_*a*_ value, such as 5-fluorouracil (5FU) and cyclophosphamide, may have increased efficacy at an acidic pH_*e*_. In contrast, the anti-tumor drugs with weak base p*K*_a_ values, such as doxorubicin, mitoxantrone and daunorubicin, may not be fully functioned because acidic pH_*e*_ reduces their cytotoxicity [121,131]. In early-stage breast cancer, high CAIX is a predictive marker of doxorubicin resistance [132].

Because *cis*-diamminedichloroplatinum (II) (CDDP) solution has an acidic pH, NaHCO_3_ is used to prevent the angialgia in the cancer patients coming from the acidic pH solution injection because it increases pH [133,134]. However, CDDP is frequently used for co-injection with other chemotherapeutic drugs, such as 5FU. In some cases, co-injection of NaHCO_3_ (depends on the concentration) may reduce the clinical efficacy of 5FU + CDDP regimen.

### Hyperthermia and acidic pH_*e*_

Hyperthermic treatment (42.5°C) for JB-1-E plasmacytoma tumor cells *in vitro* enhances the colony formation index when cells are maintained at pH 6.4, regardless oxygen tensions [135]. Melanoma cells growing at low pH are sensitized to hyperthermia because of the altered intracellular pH threshold for the heat sensitization *in vitro*[136,137].

## Conclusion

Acidic pH_*e*_ is toxic to many cells, including tumors [138]. However, if tumors have successfully adapted to their condition, and use it for their own cellular activation, this increases drug resistance and leads to more aggressive behavior. Therefore, management of tumor pH_*e*_ and inhibition of blockade of proton-sensing system are important in not only raising drug efficacy, *e.g.* mitoxantrone, but in preventing metastasis.

## Abbreviations

pHe: Extracellular pH; NHE: Na^+^/H^+^ exchanger; MCT: Monocarboxylate transporter; CA: Carbonic anhydrase; IL: Interleukin; NF-κB: Nuclear factor κB; VEGF: Vascular endothelial growth factor; HIF: Hypoxia inducible factor; LDH: Lactate dehydrogenase; OGR: Ovarian cancer G-protein-coupled receptor; PG: Prostaglandin; ASIC: Acid-sensing ion channel; KO: Knockout; FAK: Focal adhesion kinase; MMP: Matrix-metalloproteinase; iNOS: Nitric oxide synthase; PLD: Phospholipase D; MAPK: Mitogen-activated protein kinase; TNF-α: Tumor necrosis factor-α; HA: Hyaluronan; MT1-MMP: Membrane-type 1 matrix metalloproteinase; TRP: Transient receptor potential; CDDP: *cis*-Diamminedichloroplatinum (II).

## Competing interests

The authors declare no competing financial interests.

## Authors’ contributions

YK designed the study. SO, CM, YM, AS and TM were involved in discussion. YK drafted the manuscript. YB revised the manuscript. All authors read and approved the final manuscript.
